# Stability of dental implants using AnyCheck and Osstell devices

**DOI:** 10.6026/973206300210305

**Published:** 2025-03-31

**Authors:** Abhigyan Manas, Saikiran Bahadur, Tanu Priya Sonkar, Sanapala Mounika, Vaibhav Awinashe, Girish S.A, Sonali Mahajan

**Affiliations:** 1 Department of Oral & Maxillofacial Surgery, Uttar Pradesh University of Medical Sciences, Saifai, Etawah, Uttar Pradesh, India; 2 Departments of Prosthodontics, Dental Director, Springfield Dental, 4 Matthew drive, Suffield Connecticut, United States of America; 3 Department of Dentistry, Autonomous State Medical College, Firozabad, Utter Pradesh, India; 4 Intern, Kalinga Institute of Dental Sciences, Bhubaneswar, Odisha, India; 5 Department of Prosthodontics, College of Dentistry, Qassim University, Kingdom of Saudi Arabia; 6 Department of Dentistry, Siddaganga Medical College and Research Institute, Tumakuru-572102, Karnataka, India; 7 Department of Prosthodontics, Government Dental College and Hospital, Aurangabad, Maharashtra, India

**Keywords:** Dental implant, resonance frequency, stability

## Abstract

One significant element influencing the success rate of implant treatment is stability. Therefore, it is of interest to assess the stability of two distinct implant placement devices. One is based on resonance frequency analysis (Osstell®) and the other is based on damping capacity assessment (AnyCheck®). Hence, a total of 30 implants were placed for 20 patients in both maxilla and mandibular area. Primary and secondary implant stability was checked with Osstell® and AnyCheck® methods. Data shows similar performance with both the methods.

## Background:

Osseointegration is essential for an implant to be successful. One indicator of implant stability is osseointegration [1]. Osseointegration is the process by which an implant merges with the surrounding bone throughout the healing phase, giving the prosthetic tooth or crowns a secure base. The stability of the implant affects the functional loading time. Monitoring the stability of the implant during the healing phase is essential to guarantee good osseointegration and lower the risk of problems [2]. The most important factor in the effective treatment of dental implants is the stability of the implant-bone interface [3]. Primary and secondary stages are the two stages during which implant stability is measured [4]. The absence of clinical movement at the moment of implant implantation is known as the initial (primary) stability of dental implants. It has a significant impact on how well implant treatments work [5]. The mechanical coherence between the dental implant fixture and bone just after implantation is known as initial implant stability. Early implant failure had been thought to be caused by a lack of primary stability. The amount of bone, the surgical method and the implant's microscopic and macroscopic shape all affect stability [6]. Biological stability through bone remodelling and regeneration provides the secondary stability [7]. Histologic analysis, radiography, percussion testing, insertion torque, reverse torque testing and cutting torque resistance analysis are some of the techniques used to evaluate implant stability [8], but their reliability has been called into doubt in a number of investigations. Depending on the dentist's experience and skill, these techniques may have an impact on the assessment's accuracy [2, 4]. As a result, non-invasive techniques like damping capacity assessment (DCA) examples Periotest® and AnyCheck® and resonance frequency analysis (RFA) examples Osstell® were introduced [9]. To measure implant stability and osseointegration, a straightforward, reliable, non-invasive test is ideal. A scale ranging from 1 to 100 is used to denote the ISQ value. Implant stability can be evaluated using ISQ measurements. Low stability is often indicated by an ISQ score of less than 60, moderate stability is suggested by a number between 60 and 69 and high stability is indicated by a value of 70 or higher. Generally speaking, implant stability increases with an ISQ value [2]. The main way that the resonance frequency analysis-based device (Osstell®, Goteborg, Sweden) works is by creating an electric or magnetic impulse that stimulates an implant-connected transducer. The oscillation that results causes a slight lateral dislocation of the implant and the resonance frequency value is converted to the implant stability quotient (ISQ) [10]. The implant stability quotient (ISQ), which increases from 1 to 100 as implant stability increases, is the OsstellTM measured value [11].

The DCA approach measures the implant's fluctuation in both longitudinal and lateral directions after applying a specific amount of force mechanically to the implant post [2]. A DCA tool called Periotest® (Medizintechnik Gulden, Germany) was first created to quantify tooth movement [12]. The Periotest® uses an accelerometer to determine how long it takes for the tapping head to touch a tooth or implant. The results are presented as Periotest values (PTVs), which can be anywhere, from -8 to +50. Higher stability is indicated with lower numbers [13]. The DCA approach measures the implant's fluctuation in both longitudinal and lateral directions after applying a specific amount of force mechanically to the implant post [2]. A damping capacity assessment tool called Periotest® (Medizintechnik Gulden, Germany) was first created to quantify tooth movement [12]. The Periotest® uses an accelerometer to determine how long it takes for the tapping head to touch a tooth or implant. The results are presented as Periotest values (PTVs), which can be anywhere, from -8 to +50. Higher stability is indicated with lower numbers [13]. By refining the striking technique, it assesses stability by direct contact with the item. By timing the striking rod's (head) contact with the implant or abutment, this device assesses the osseointegration between the implant and alveolar bone. The Implicit Stability Test (IST) value, which is a number between 1 and 99, is the measurement that was acquired from the modified device. Classifying implant stability is aided by the colour-coding of IST values [2]. Therefore, it is of interest to assess implant stability using two distinct devices based on damping capacity assessment (AnyCheck®) and resonance frequency analysis (Osstell®).

## Materials and Methods:

## Study design:

This research was done in the Oral and Maxillofacial Surgery department after obtaining the approval from the concerned authority and consent from all the participants. The study was done by trained investigator from March 2023 to October 2024. Total 20 healthy patients of both genders who require one or more implant placement for missing teeth were included for the study. Total 30 implants were placed for 20 patients in both maxilla and mandibular area. Following dental cone-beam computed tomography (CBCT), the quantity and quality of bone were assessed in order to choose and position an optimal implant. Neobiotech's CMI IS-II implant, located in Seoul, the Republic of Korea, was utilised. 8.5/10.0/11.5 mm in length and 3.5/4.0/4.5/5.0 mm in diameter were employed in this investigation. Both the RFA (Osstell®) and DCA (AnyCheck®) methods were used to determine the primary (baseline) implant stability at the time of insertion and the secondary stability four weeks later. An Osstell1 Beacon+ (Integration Diagnostics, Göteborg, Sweden) was used to perform the RFA measurements. The smart peg was manually attached to the implant fixture in order to take measurements using the Osstell ISQ Mentor. Every gadget was operated in compliance with the guidelines provided by the manufacturer. According to the Osstell ISQ Mentor's manufacturer, the device tip should be held at a 45-degree angle and close (2.0-4.0 mm) to the smart peg top without contacting it. Primary and secondary stages are the two stages during which implant stability is measured [4]. The absence of clinical movement at the moment of implant implantation is known as the initial (primary) stability of dental implants. It has a significant impact on how well implant treatments work [5]. The mechanical coherence between the dental implant fixture and bone just after implantation is known as initial implant stability. Both devices' main and secondary implant stability were examined and contrasted. For both devices, stability was examined at insertion torques of greater than 50 N/cm and less than 50 N/cm. After attaching a 4 mm high healing abutment to the implant with a standardised torque of 20 N/cm, the RFA and DCA equipment took the measurement. Following the Osstell ISQ Mentor and DCA technique measurements, the implant was fitted with healing abutments (Neobiotech, Seoul and Republic of Korea). The obtained data was statistically evaluated using SPSS software version 23.0 using t test, Mann Whitney U test at P<0.05.

## Results:

ISQ-Implant stability quotient and IST-Implant stability test result shown in Table 1. According to Table 1, the dental implant that was inserted with an insertion torque of more than 50 N/cm showed greater primary and secondary stability as determined by both devices than the dental implant that was implanted with an insertion torque of less than 50 N/cm. Osstell® and AnyCheck® showed a substantial positive correlation for the primary stability. In contrast, both devices showed high correlations with comparable patterns for secondary stability (Table 2). The overall mean ISTs ranged from 0 to -7 with an average value of -4.24 and a standard deviation of 1.28 and ISQ values were 71.76 ± 3.18 (range, 57 to 85). There was a very strong negative correlation between mean IST and ISQ values in first measurements r = -0.953 P = 0.001 as well as in the second measurements r = -0.903, P = 0.001 (Table 3).

## Discussion:

A stable implant is essential for a successful osseointegration process. For dental implant insertion to produce excellent clinical results, implant stability must be maintained. Assessing implant stability helps make the right decisions regarding loading status, enables patient-by-patient selection of appropriate protocols, identifies situations in which it is better not to load, builds patient trust, promotes effective communication and improves case documentation [14]. The two devices in the current investigation exhibited moderate to significant correlations in the primary and secondary stability measurements. Implant stability and PTV have an inverse relationship; low levels indicate good stability, whereas high values indicate poor stability.

Effective osseointegration and "good stability" are indicated by PTVs between -8 and 0, "moderate stability" by values between 1 and 9 and "poor stability" by values between 10 and 50 [15]. In the literature, the average PTV for osseointegrated implants varied between -8 and ±5.5 [16]. According to Andersson *et al.* mechanical relaxation and/or bone remodelling in response to high pressures experienced during dental implant insertion may be the cause of the gradual decline in initial implant stability [17]. Osstell is a good tool for evaluating implant stability, according to Parmar *et al.* [18]. For osstell and periotest devices, Kocak-Buyukdere *et al.* discovered a high positive association between the mean percentage changes of PTV and ISQ values [19]. Our findings are consistent with the results. Dhahi *et al.* used three instruments (Osstell®, Periotest® and AnyCheck®) to measure the primary and secondary stabilities of implants and came to the conclusion that all three are accurate [5]. These outcomes are consistent with what we found. Motea used five analytical tests insertion torques, removal torques; resonance frequency analysis, push-in test and pull-out test-to assess the initial stability of a dental implant with a horizontal plate. They came to the conclusion that, in comparison to dental implants without horizontal plates, those with them exhibited superior primary stability [6]. After comparing the early implant stability results from resonance frequency analysis (RFA) and damping capacity analysis (DCA) using the in vitro approach, Lee *et al.* came to the conclusion that Anycheck® was simpler and easier to use than the Osstell® Beacon+ [2]. According to Ji-Suk Shim's research, Anycheck demonstrated relative reliability in comparison to Periotest M and Osstell ISQ Mentor [20].

Anycheck is helpful in assessing implant stability, according to Okuhama *et al.*'s comparison of the osstell and Anycheck for stability measurement [21]. Our findings are consistent with these results. Depending on the patient's position and the implant's placement, Lee *et al.* assessed the accuracy of implant stability measurement devices. They came to the conclusion that the instruments used to measure implant stability are less reliable. In the order of Osstell, Anycheck and Periotest, the accessibility of implants is significantly impacted [22]. The current study demonstrates the efficacy of damping capacity assessment (AnyCheck®) and resonance frequency analysis (Osstell®) in identifying implant stability.

## Conclusion:

Data shows that resonance frequency analysis (Osstell®) and the other are based on damping capacity assessment (AnyCheck®) are trustworthy methods for determining implant stability.

## Figures and Tables

**Table 1:** The association among the primary and secondary dental implant insertion torque and stability

**Device used**	**Primary stability**		**p**	**Secondary stability**		**p**
	**Insertion torque N/cm**	**Mean±SD**		**Insertion torque N/cm**	**Mean ±SD**	
Osstell/ISQ	<50	71.71±.6	0.001*	<50	70.54±3.5	0.031*
	>50	74.62±2.4		>50	74.22±.2	
AnyCheck/IST	<50	71.51±3.7	0.021*	<50	72.31±3.2	0.001*
	>50	74.83±3.5		>50	75.21±3.4	
*-significant Test used-Mann Whitney U test

**Table 2:** The association of both devices for primary and secondary dental implant stability

**Device used**	r	**p**
**Primary stability measurements**		
Osstell/ISQ vs. AnyCheck/IST	0.6	<0.0001*
Secondary stability measurements		
Osstell/ISQ vs. AnyCheck/IST	0.8	<0.0001*

**Table 3:** The relationship among IST and ISQ values in first and second measurements

		**Mean IST baseline**	**Mean IST baseline**
Mean ISQ	R	-0.953**	-0.714**
Baseline	P	0	0
	N	51	51
Mean ISQ	R	-0.745**	-0.903**
Baseline	P	0	0
	N	51	51
